# Assessing the built environment through photographs and its association with obesity in 21 countries: the PURE Study

**DOI:** 10.1016/S2214-109X(24)00287-0

**Published:** 2024-09-27

**Authors:** Daniel J Corsi, Simone Marschner, Scott Lear, Perry Hystad, Annika Rosengren, Rosnah Ismail, Karen Yeates, Sumathi Swaminathan, Thandi Puoane, Chuangshi Wang, Yang Li, Sumathy Rangarajan, Iolanthé M Kruger, Jephat Chifamba, K Vidhu Kumar, Indu Mohan, Kairat Davletov, Galina Artamonov, Lia M Palileo-Villanueva, Nafiza Mat-Nasir, Katarzyna Zatonska, Aytekin Oguz, Ahmad Bahonar, Khalid F Alhabib, Afzalhussein Yusufali, Patricio Lopez-Jaramillo, Fernando Lanas, Agustina Galatte, Álvaro Avezum, Martin Mckee, Salim Yusuf, Clara K Chow, Prospective Urban Rural Epidemiology (PURE) Study Investigators

**Affiliations:** a School of Epidemiology and Public Health, Faculty of Medicine, University of Ottawa, Ottawa, ON, Canada; b Westmead Applied Research Centre, Faculty of Medicine & Health, The University of Sydney, Westmead, NSW, Australia; c Faculty of Health Sciences, Simon Fraser University, Burnaby, BC, Canada; d School of Biological and Population Health Sciences, College of Public Health and Human Sciences, Oregon State University, Corvallis, OR, USA; e Sahlgrenska Academy, University of Gothenburg and Sahlgrenska University Hospital, Gothenburg, Sweden; f Department of Community Health, Faculty of Medicine, University Kebangsaan Malaysia, Bangi, Malaysia; g Department of Medicine, Queen's University, Belfast, UK; h St John's Research Institute, Bangalore, India; i School of Public Health, University of the Western Cape, Cape Town, South Africa; j Medical Research and Biometrics Center, National Center for Cardiovascular Disease, Fuwai Hospital, Beijing, China; k Population Health Research Institute, Hamilton Health Sciences, McMaster University, Hamilton, ON, Canada; l Africa Unit for Transdisciplinary Health Research, North-West University, Potchefstroom, South Africa; m Department of Biomedical Sciences, University of Zimbabwe, Harare, Zimbabwe; n Health Action by People, Kerala, India; o Mahatma Gandhi University of Medical Sciences and Technology, Jaipur, India; p Asfendiyarov Kazakh National Medical University, Almaty, Kazakhstan; q Federal State Budgetary Institution Research Institute for Complex Issues of Cardiovascular Diseases, Kemerovo, Russia; r UP College of Medicine, University of the Philippines Manila, Manila, Philippines; s Department of Primary Care Medicine, Faculty of Medicine, Universiti Teknologi MARA (UiTM), Selangor, Malaysia; t Department of Population Health, Wroclaw Medical University, Wroclaw, Poland; u Department of Internal Medicine, Faculty of Medicine, Istanbul Medeniyet University, Istanbul, Türkiye; v Isfahan Cardiovascular Research Center, Cardiovascular Research Institute, Isfahan University of Medical Sciences, Isfahan, Iran; w Department of Cardiac Sciences, King Fahad Cardiac Center, College of Medicine, King Saud Medical City, King Saud University, Riyadh, Saudi Arabia; x Tamani Foundation, Zanzibar, Tanzania; y Masira Research Institute, Universidad de Santander, Bucaramanga, Colombia; z Universidad de La Frontera, Temuco, Chile; aa ECLA, Rosario, Santa Fe, Argentina; ab International Research Center, Hospital Alemão Oswaldo Cruz, São Paulo, Brazil; ac London School of Hygiene & Tropical Medicine, London, UK

## Abstract

**Background:**

The built environment can influence human health, but the available evidence is modest and almost entirely from urban communities in high-income countries. Here we aimed to analyse built environment characteristics and their associations with obesity in urban and rural communities in 21 countries at different development levels participating in the Prospective Urban and Rural Epidemiology (PURE) Study.

**Methods:**

Photographs were acquired with a standardised approach. We used the previously validated Environmental Profile of a Community's Health photo instrument to evaluate photos for safety, walkability, neighbourhood beautification, and community disorder. An integrated built environment score (ie, a minimum of 0 and a maximum of 20) was used to summarise this evaluation across built environment domains. Associations between built environment characteristics, separately and combined in the integrated built environment score, and obesity (ie, a BMI >30kg/m^2^) were assessed using multilevel regression models, adjusting for individual, household, and community confounding factors. Attenuation in the associations due to walking was examined.

**Findings:**

Analyses include 143 338 participants from 530 communities. The mean integrated built environment score was higher in high-income countries (13·3, SD 2·8) compared with other regions (10·1, 2·5) and urban communities (11·2, 3·0). More than 60% of high-income country communities had pedestrian safety features (eg, crosswalks, sidewalks, and traffic signals). Urban communities outside high-income countries had higher rates of sidewalks (176 [84%] of 209) than rural communities (59 [28%] of 209). 15 (5%) of 290 urban communities had bike lanes. Litter and graffiti were present in 372 (70%) of 530 communities, and poorly maintained buildings were present in 103 (19%) of 530. The integrated built environment score was significantly associated with reduced obesity overall (relative risk [RR] 0·58, 95% CI 0·35–0·93; p=0·025) for high compared with low scores and for increasing trend (0·85, 0·78–0·91; p<0·0001). The trends were statistically significant in urban (0·85, 0·77–0·93; p=0·0007) and rural (0·87, 0·78–0·97; p=0·015) communities. Some built environment features were associated with a lower prevalence of obesity: community beautification RR 0·75 (95% CI 0·61–0·92; p=0·0066); bike lanes RR 0·58 (0·45–0·73; p<0·0001); pedestrian safety RR 0·75 (0·62–0·90; p=0·0018); and traffic signals RR 0·68 (0·52–0·89; p=0·0055). Community disorder was associated with a higher prevalence of obesity (RR 1·48, 95% CI 1·17–1·86; p=0·0010).

**Interpretation:**

Community built environment features recorded in photographs, including bike lanes, pedestrian safety measures, beautification, traffic density, and disorder, were related to obesity after adjusting for confounders, and stronger associations were found in urban than rural communities. The method presents a novel way of assessing the built environment's potential effect on health.

**Funding:**

Population Health Research Institute, Hamilton Health Sciences Research Institute, Heart and Stroke Foundation of Ontario, Canadian Institutes of Health Research's Strategy for Patient Oriented Research, Ontario Support Unit, Ontario Ministry of Health and Long-Term Care, AstraZeneca, Sanofi–Aventis, Boehringer Ingelheim, Servier, and GlaxoSmithKline.

## Introduction

The concept of healthy cities is based on growing evidence that the built environment can affect cardiometabolic health outcomes.^1^ Several studies and systematic reviews have shown associations between built environment measures, particularly measures of a neighbourhood's walkability, and health outcomes, such as obesity, diabetes, and hypertension.^2^, ^3^, ^4^, ^5^, ^6^ Communities in the USA, Canada, and Australia with high walkability scores, indicating a built environment amenable to walking, have shown lower levels of BMI and obesity than communities with low walkability scores.^7^, ^8^, ^9^, ^10^, ^11^ Studies have also shown links between the built environment and intermediary behaviours, including physical activity.^12^ These findings have also been replicated using study designs that incorporate changes over time and adjustments for confounding.^13^, ^14^, ^15^

**Research in context**

**Evidence before this study**

On March 25, 2024, we searched PubMed for papers published in English from database inception using the search terms “built environment” OR “physical environment” OR “walkability” AND “health” or “cardiovascular disease” or “obesity” or “adiposity” or “overweight” or “diabetes” or “hypertension” along with the reference lists of identified publications. The evidence suggests that the built environment might influence cardiovascular health. However, evidence is mixed in quality and consistency. In addition, most studies are based in urban communities in high-income countries.

**Added value of this study**

To the best of our knowledge, this study is the first to estimate built environment characteristics through photographs in diverse communities. In addition, it shows the association between these characteristics and BMI and obesity in a cohort of individuals from urban and rural communities in countries at different economic levels participating in the Prospective Urban and Rural Epidemiology study. It shows that greater traffic density and community disorder levels were associated with obesity. At the same time, certain features of the built environment, including bike lanes, pedestrian safety features, and beautification, were inversely associated with obesity.

**Implications of all the available evidence**

Most research that measures the built environment either uses comprehensive time-taking direct audit techniques or secondary data sources—often not comparable between countries. These approaches limit the examination of the built environment and its relation to health in multicentre international studies. This study shows a scalable and replicable method to assess the built environment across diverse communities. We further show that these built environment measures are associated with obesity among diverse regions of the world and that the strength of the relationship differs by community type (ie, stronger in urban compared with rural communities). Our findings suggest that this method could be used to track the built environment health of communities and, in the future, improve our understanding of how communities change their built environment and how this impacts health.

However, there are few multicountry studies, especially ones including low-income countries.^16^ Given that published studies on the built environment were done in high-income settings, there is a need to confirm the associations with health using data from other settings, such as rural areas or low-income countries, to understand whether the associations are causal or context-specific. The dearth of data from low-resourced regions is an important incentive for this research. A major barrier to research in low-income and middle-income countries is the difficulty in quantifying the built environment and linking this information to health outcomes. Built environment assessments frequently rely on secondary data captured based on organisational units (eg, streets and postcodes), often consistently recorded within a city or a country but not between countries. Secondary data could have suboptimal validity or other limitations. Also, built environment assessment commonly uses geographical information systems, which are typically less comprehensive and less consistently available in low-income settings than high-income settings.^17^ Studies in high-income countries now use routinely collected street-view data, such as Google Street View images, to assess built environment features, with some applications in middle-income countries (especially China).^18^, ^19^ Such images are only partly available in countries outside North America and Europe and more coverage of rural areas in low-income and middle-income countries is needed. The lack of deployment of traditional built environment data collection methods in low-income settings is unlikely to change in the short term. As a result, a need exists for pragmatic and reliable approaches to collecting built environment data in various contexts, including low-resourced regions.

The Prospective Urban Rural Epidemiology (PURE) study uses standardised methods to collect reported and measured data on cardiometabolic risk factors among community-based cohorts of adults from a diverse range of low-income, middle-income, and high-income communities and countries.^20^, ^21^ The PURE design allows for comparison across countries and enables built environment data collection in low-income and middle-income countries. As existing neighbourhood audit tools^22^, ^23^, ^24^, ^25^ were not applicable or feasible to implement consistently across the wide range of communities in PURE, we designed and validated the Environmental Profile of a Community's Health (EPOCH) tool.^26^, ^27^ We also developed a built environment photo-capture instrument (EP-NET) to create standardised measures of the environment that were potentially related to obesity and physical activity.^28^ We hypothesised that there are significant differences in built environment attributes across communities from different regions of the world and that these attributes are associated with obesity and intermediate behaviours such as walking. Here, we aim to compare built environment features across communities from different regions of the world and examine the associations between these attributes and obesity and walking and determine whether they were consistent in urban and rural communities, across regions and by country economic wealth status.

## Methods

### Study design

We assessed PURE communities and collected built environment data using photographs between 2008 and 2019.^26^, ^27^, ^28^, ^29^ The PURE study is an ongoing community-based prospective cohort survey that has enrolled more than 202 000 adults aged 35–70 years at baseline in several waves from urban and rural communities in 27 low-income, lower-middle-income, upper-middle-income, and high-income countries.^30^, ^31^ We selected urban and rural communities from different countries using a stratified non-random approach. Communities were selected to achieve broad representativeness of demographic and socioeconomic profiles and area-level characteristics.^32^ The Hamilton Integrated Research Ethics Board approved the PURE and EPOCH studies (REB Project number 03-206) along with local institutional research ethics boards in participating countries.

### Individual data collection

Within communities, a representative sample of adults aged 35–70 years was enrolled by approaching all households or a random sample of households to participate as determined by local investigators. Health histories and clinical and anthropometric data were collected at baseline using questionnaires and physical exams by trained research staff. These included sociodemographic data (eg, age, sex, education, and household wealth), cardiovascular risk factors (eg, smoking status, history of hypertension, diabetes, and cardiovascular disease), and physical measurements (eg, weight, height, blood pressure, and waist circumference). In addition, the International Physical Activity Questionnaire was added to the study and completed by individuals recruited after 2008.^30^

### Built environment data collection

The PURE cohort was established around a community-level recruitment strategy through which individual participants from defined geographical communities were invited to participate. Selected communities in which recruitment occurred were later assessed to profile environmental features, and photographs of each community were taken.^26^ We collected a standardised set of photographs from 530 PURE communities. Local researchers were provided with an operation manual and training webinars on collecting photographic data by the central study team in Canada. To standardise the photo data collection, we trained data collectors to optimise lighting, angles, and views and how to overlap images to obtain a 360-degree view with complete coverage of street scenes from a designated central start point. We used a central intersection of an area to photograph, and researchers collected all photographic data from this location during daylight hours. We then developed the EP-NET photo tool to analyse and extract built environment data from collected photographs.^28^ The items included in EP-NET were drawn from our literature review, focusing on features of the community environment related to walkability and could be identified from photographs.^33^ Two reviewers independently scored photos collected from each community to validate the EP-NET instrument. Validation data show high reliability and reproducibility, 53 (88%) of 60 items had an intra-class correlation coefficient greater than 0·7.^28^

### Outcomes

The primary outcome was obesity (ie, a BMI ≥30 kg/m^2^) measured at baseline. The standard BMI threshold was selected for comparability and consistency across regions, and we did not use variable cutoffs by ethnicity or country. Our secondary outcome was physical activity from walking. We selected walking physical activity as an intermediate outcome and general indication of physical activity across the PURE communities. Walking was measured in metabolic equivalents (MET)-minutes (MET × min per week). Walking MET minutes were calculated from the long-form International Physical Activity Questionnaire data as the number of min walking per week multiplied by the MET value for walking.^34^

### Exposures

EP-NET includes 37 discrete items, scored centrally for each community by the research team using available photographs. In this analysis, we selected 32 items from EP-NET that objectively assessed the absence or presence of a feature (eg, sidewalks), a direct count (eg, the number of bicycles and cars seen), or a semi-quantitative count (eg, none or one or two, some, or many) of features. Five items from the EP-NET instrument related to the subjective assessment of neighbourhood appeal and satisfaction were excluded. We aggregated each separate item into summary scales, representing pedestrian safety features (eg, sidewalks [pedestrian-only walkways alongside roads], crosswalks [marked places for pedestrians to cross roads], and traffic signals), bicycle safety (eg, bike lanes), traffic density (ie, numbers of cars), community beautification (meaning positive aesthetic features such as landscaping, grass, flowers, trees, public art, benches, and lighting) or natural features (eg, mountains, bodies of water, and forest), and community disorder (eg, the presence of litter, graffiti, and poorly maintained buildings), used in all analyses (panel). An integrated built environment score was constructed by summing these scales, with reverse coding used for scales positively associated with obesity (eg, traffic density and disorder). The rationale for developing the integrated score as an overall summary of the built environment was that combining these features better captures the underlying concept of an improved built environment. Individual participants in PURE were then matched directly to the photographic data obtained from their communities.

Panel **Features of the built environment identified through photographs in the EPOCH Photos Neighbourhood Evaluation Tool**

**Pedestrian safety (minimum score 0–maximum score 14)**

• Sidewalks (0–1)
• Sidewalk completeness (0–2)
• Sidewalk quality (0–3)
• Crosswalks (0–1)
• Crosswalk quality (0–5)
• Median strips (0–2)

**Traffic signals (0–1)**

• Traffic signals with pavement markings (0–2)*

**Community beautification (0–12)**

• Natural features (0 if no, 1 if yes to field, body of water, mountain, green belt, forest, or desert)
• Street trees (0–3)
• Man-made landscaping (0–3)
• Street furniture (eg, benches, trash cans, bus shelters, and streetlamps; 0 if no, 1 to 4 if yes to benches, rubbish bins, bus shelters, streetlamps)
• Public art (0–1)

**Community disorder (0–4)**

• Litter or rubbish present (0–1)
• Graffiti present (0–1)
• Derelict buildings (0–1)
• Buildings poorly maintained (0–1)

**Traffic density (0–9)**

• Street density (0–3)
• Vehicle density (0–3)
• Parked cars (0–3)

**Bike lanes (0–1)**

• Presence of dedicated cycling lanes (0–1)

**Integrated Built Environment Score** † **(4–20)**

• Sum of beautification (1–6)
• Disorder (1–4)
• Traffic density (1–4)
• Bike lanes (0–1)
• Pedestrian safety (1–4)
• Traffic signals (0–1)

EPOCH=Environmental Profile of a Community's Health.

### Covariates

Our adjusted analyses included age, sex, education, household wealth, country income classification, urban–rural location, and community-level wealth as a marker of socioeconomic status. Age was modelled in single years; sex was self-reported and dichotomised as male or female. Education was categorised as none or primary only, versus secondary or higher secondary, versus trade school, college, or university. Household wealth was based on an index of ownership of assets and housing characteristics. The descriptors of economic level were established at study initiation based on World Bank data from 2006 and are the country income classification maintained in all PURE research papers.^35^ Urban and rural communities were classified using country-specific criteria.^20^ Household wealth scores were aggregated to the community level as a proxy for the community standard of living and income and included as a potential confounder. Walking physical activity was also used in regression models to account for physical activity as a covariate and to examine the attenuation in effect estimates of the built environment features on obesity when including walking as a marker for physical activity.

### Statistical analysis

We describe the built environment features from the photograph assessment using frequencies and percentages for categorical variables and means and standard deviations for continuous variables. Descriptive analyses are presented separately for urban and rural areas and by country economic development level.

Scales representing built environment features were collapsed so that features were modelled from low to high with a maximum of six categories. We defined quartiles for the integrated built environment score based on the total number of built environment features observed. We constructed a directed acyclic graph depicting the relationships between the built environment attributes (exposures), outcomes, and covariates (appendix p 8). We use this modelling framework for subsequent statistical analyses. Built environment attributes were related to obesity using multilevel Poisson regression models with a log link and robust error variance.^36^ This model is an alternative to logistic regression for frequently observed binary outcomes, for which the odds ratio might overestimate the prevalence ratio. The Poisson model can directly estimate the prevalence ratio; we report this as the relative risk (RR). Models included random intercepts for communities and were adjusted for age, sex, country-income level, individual-level education, urban–rural location, household wealth, and community-level wealth. Built environment exposures were modelled in two ways. First, they were measured continuously as a linear trend of the increasing built environment score on obesity. Second, we used categories to assess the association of different built environment levels with outcomes without assuming linearity in the exposure–outcome relationship. In these models, the lowest category of built environment measure was treated as the reference. Each model includes a single built environment attribute adjusted for covariates. We used an identical approach for our secondary outcome of walking physical activity (in MET-minutes), replacing the Poisson model with a linear multilevel model.

Our secondary analyses included region-specific models in which we examined the association between the integrated built environment score and obesity, and these models did not include country-income level. To explore the effect of physical activity on our estimates, we fitted a fully adjusted model, including walking physical activity as a potential mediator and a second model without walking. We assessed attenuation in the association between built environment features and obesity after including physical activity from walking in the models using the formula, attenuation %=(1–RR_2_/RR_1_) × 100, where RR_2_ is the RR from the adjusted model including walking and RR_1_ is from the model without walking.

We tested association heterogeneity between the built environment features and outcomes using interaction effects for urban–rural location, country-income level, and geographical region (ie, south Asia, China, southeast Asia, Russia and former Soviet Republics, North America and Europe, the Middle East, and South America). We present the joint p value from these analyses, representing the combined heterogeneity test across all categories. We present the overall analyses and stratified analyses by urban–rural location. We compared built environment measures from the EPOCH photographs with measures from the Neighbourhood Environment Walkability Scale,^37^ collected from a subset of respondents in the PURE study. This validated self-reported measure asked respondents about their perception of built environment domains, including street connectivity, neighbourhood aesthetics, walking infrastructure, and safety from traffic. We examined the concordance between overlapping domains between the two measures using Spearman correlations. Statistical tests were two-sided with an alpha of 0·05. All statistical analyses were performed with Stata (version 17.0/MP) and models estimated with the multilevel mixed effects generalised linear models procedure.

### Role of the funding source

The funders of the study had no role in study design, data collection, data analysis, data interpretation, or writing of the report.

## Results

Complete photographic data were available for 530 (53%) of 998 PURE communities (ie, 112 from high-income countries [HICs], 143 from upper-middle-income countries [UMICs], 188 from lower-middle-income countries [LMICs], and 87 from low-income countries [LICs]) and BMI measurements were available from 143 338 individuals from these communities (appendix p 2). 290 (55%) of 530 communities were urban. The mean age of participants was 50·8 years (SD 9·9), and 84 559 (59·0%) of 143 338 participants were female (table 1). The mean BMI was 25·9 (SD 5·3) kg/m^2^, with substantial variation by country (appendix p 9). The prevalence of obesity was 18·6% (95% CI 18·4–18·8); it was higher in females (21·3%, 21·0–21·5) than males (14·9%, 14·6–15·1) and in those with lower education (20·4%, 20·1–20·8) compared with higher education (16·4%, 16·1–16·7; appendix p 3). In LICs, LMICs, and UMICs, obesity was more frequent in urban areas (20·2%, 19·9–20·5) than in rural areas (14·5%, 14·2–14·8). However, in HICs, rural areas had higher rates of obesity (appendix p 3).

**Table 1:** Demographic characteristics of the PURE cohort (N=143 338)

		**High-income countries**				**Upper-middle-income countries**				**Lower-middle-income countries**				**Low-income countries**			
		Urban (n=12 355)		Rural (n=4508)		Urban (n=21 977)		Rural (n=18 790)		Urban (n=29 718)		Rural (n=27 835)		Urban (n=14 145)		Rural (n=14 010)	
		n or n (%)	SD	n or n (%)	SD	n or n (%)	SD	n or n (%)	SD	n or n (%)	SD	n or n (%)	SD	n or n (%)	SD	n or n (%)	SD
Sex																	

	Female	6587 (53·3%)	··	2466 (54·7%)	··	13 777 (62·7%)	··	11 175 (59·5%)	··	17 910 (60·3%)	··	16 308 (58·6%)	··	8070 (57·1%)	··	8266 (59·0%)	··

	Male	5768 (46·7%)	··	2042 (45·3%)	··	8200 (37·3%)	··	7615 (40·5%)	··	11 808 (39·7%)	··	11 527 (41·4%)	··	6075 (42·9%)	··	5744 (41·0%)	··

Mean age, years		51·9	9·5	53·3	9·6	51·6	9·7	51·2	9·9	51·8	9·8	49·7	9·5	49·2	10·4	48·6	10·7

Mean BMI, kg/m^2^		27·6	5·5	28·3	5·4	28·5	5·7	27·6	5·8	25·6	4·1	24·7	4·1	25·1	4·9	21·5	4·6

Trade, college, or university educated		7789 (63·0%)	··	2103 (46·7%)	··	6798 (30·9%)	··	2013 (10·7%)	··	8423 (28·3%)	··	1236 (4·4%)	··	3381 (23·9%)	··	494 (3·5%)	··

Highest household wealth level*		4569 (37·1%)	··	1461 (32·5%)	··	9520 (43·5%)	··	4933 (26·4%)	··	16 487 (56·1%)	··	3276 (11·9%)	··	7536 (57·4%)	··	1899 (15·2%)	··


* Highest tertile of household wealth, based on an index of household possessions and assets (n=140 004).

**Figure 1:**
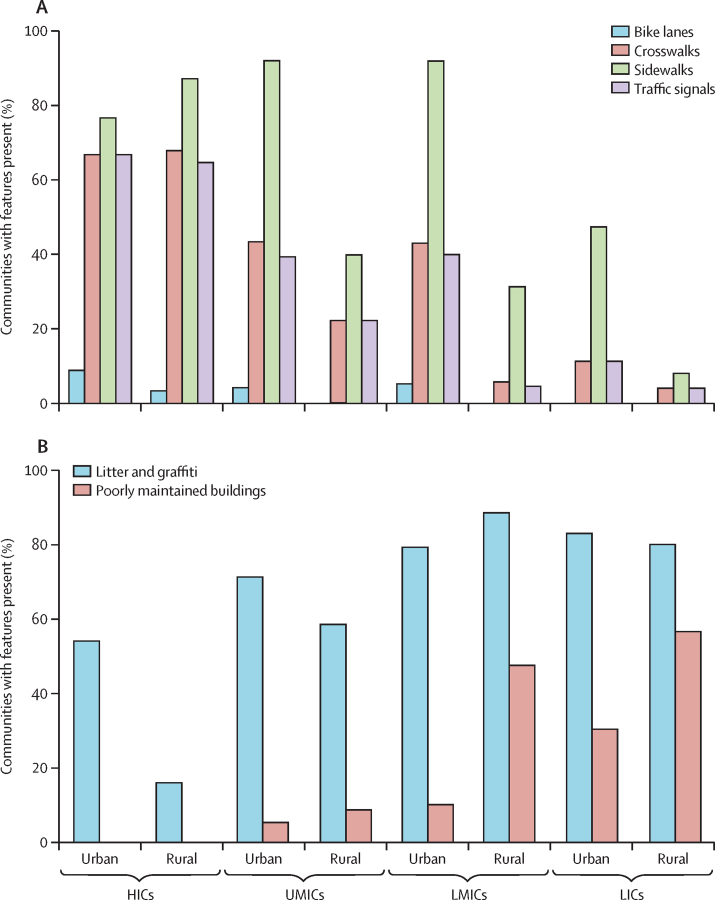
Presence of built environment features that potentially influence obesity in communities by urban–rural location and country income in 530 communities (A) Includes features of the built environment potentially associated with reducing obesity. (B) Includes features potentially associated with increasing obesity. HICs=high-income countries. LICs=low-income countries. LMICs=lower-middle-income countries. UMICs=upper-middle-income countries.

Pedestrian safety features (eg, crosswalks, sidewalks, and traffic signals) were present in a majority (>60%) of communities in HICs, with similar rates in urban and rural areas (figure 1). Elsewhere, these features were much more common in urban compared with rural areas—for example, sidewalks were found in 176 (84%) of 209 urban communities compared with 59 (28%) of 209 rural communities outside HICs. Bike lanes were infrequent, found in 15 (5%) of 290 urban communities, and sparse in rural communities. Natural features (eg, the presence of fields, bodies of water, forested areas, or other features) were common and found in most communities in HICs (102 [91%] of 112), UMICs (132 [92%] of 143), and LMICs (169 [90%] of 188) but slightly less common in LICs (70 [80%] of 87; appendix p 10). Moderate urban–rural differences were observed. Natural features were slightly more common in urban areas than rural areas, except in HICs, where they were more common in rural areas (appendix p 10).

Community disorder features such as litter and graffiti were identified in 372 (70%) of 530 communities, and poorly maintained buildings in 103 (19%) communities (figure 1). Litter and graffiti were observed in 229 (83%) of 275 urban and rural communities in LICs and LMICs and five (16%) of 31 rural and 44 (54%) of 81 urban communities in HICs. There were higher rates of poorly maintained and derelict buildings in rural LICs (29 [57%] of 51) and rural LMICs (43 [48%] of 90) compared with urban areas in both regions (21 [16%] of 134), but these features were not found in HICs.

**Figure 2:**
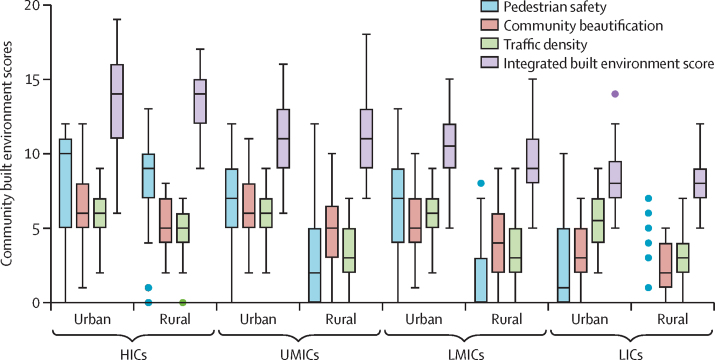
Distribution of Built Environment Feature Scores derived through community photographs, by urban–rural location and country income in 530 communities The box plots show the distribution of Built Environment Scores across urban and rural communities by country level income. The boxes indicate first and third quartiles with median lines at the centre. The whiskers indicate the first quartile=1·5 × IQR and the third quartile + 1·5 × IQR. Values outside the whiskers are shown as dots. Data from the Environmental Profile of a Community's Health Photo and Built Environment Photo-Capture scoring instrument. HICs=high-income countries. LICs=low-income countries. LMICs=lower-middle-income countries. UMICs=upper-middle-income countries.

On average, HICs had greater integrated built environment scores, with a mean score of 13·3 (SD 2·8) versus 10·1 (2·5) in non-HIC regions (figure 2). The distribution of integrated built environment scores showed a decrease from high-income urban areas to low-income rural areas. The score distributions for pedestrian safety, community beautification, and traffic density across country income level and urban rural locations are also shown in figure 2. Pearson correlations between components of the integrated built environment score were generally consistent overall and in urban and rural areas (appendix p 11).

**Figure 3:**
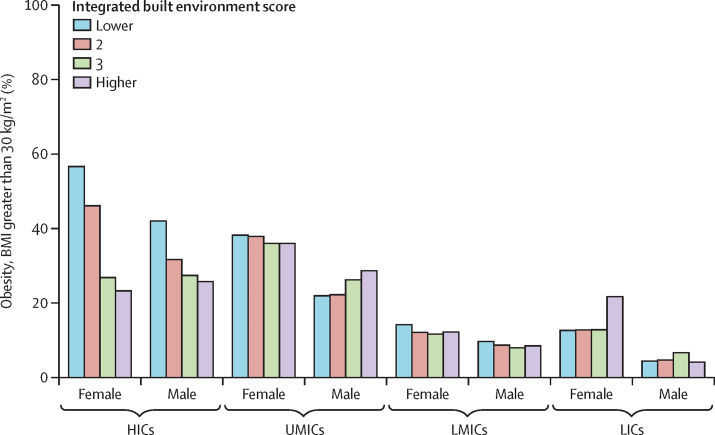
The prevalence of obesity (BMI >30 kg/m^2^) across levels of the integrated built environment score by sex and country income level HICs=high-income countries. LICs=low-income countries. LMICs=lower-middle-income countries. UMICs=upper-middle-income countries.

Descriptively, obesity was more common in communities with lower integrated built environment scores, with this pattern most evident in both males and females in HICs but less consistent in other settings. For example, an increasing trend for greater rates of obesity with higher scoring-built environments was seen among males in UMICs, and the relationship was less consistent in LICs (figure 3).

Adjusted regression analyses indicated that higher levels of pedestrian safety were associated with lower obesity in the PURE cohort overall (RR 0·75, 95% CI 0·62–0·90) and in urban areas (0·73, 0·60–0·90) but not in rural areas (1·20, 0·99–1·45; p value for heterogeneity <0·0001; table 2). Bike lanes were also associated with lower levels of obesity overall (RR 0·58, 95% CI 0·45–0·73) and in urban and rural areas (p value=0·0603). Greater community beautification was associated with lower rates of obesity overall (RR 0·75, 95% CI 0·61–0·92) and in urban areas (0·63, 0·48–0·82), but not in rural areas (0·99, 0·72–1·37; p value for heterogeneity p<0·0001). Higher levels of community disorder were associated with higher rates of obesity overall (RR 1·48, 95% CI 1·17–1·86), which was similar in urban and rural areas with some evidence of urban–rural heterogeneity (p=0·022). Higher traffic density was not associated with obesity in the overall cohort (0·99, 0·81–1·20). Trend analyses suggested that traffic density could be inversely associated with obesity in urban areas (RR for linear trend 0·85, 95% CI 0·79–0·92) but positively associated with obesity in rural areas (1·16, 1·06–1·27; p value for heterogeneity <0·0001).

**Table 2:** Associations between community built environment features and obesity (BMI >30 kg/m^2^) from fully adjusted multilevel Poisson regression models

		**Overall**			**Urban**			**Rural**			**p**_interaction_		
		RR	95% CI	p value	RR	95% CI	p value	RR	95% CI	p value	Urban–rural	Country income	Region
Pedestrian safety		··	··	··	··	··	··	··	··	··	<0·0001	<0·0001	<0·0001

	Trend	0·90	(0·85–0·95)	0·0002	0·90	(0·85–0·96)	0·0018	1·02	(0·95–1·11)	0·55	··	··	··

	Low	1·00	··	··	1·00	··	··	1·00	··	··	··	··	··

	2	0·77	(0·64–0·94)	··	0·84	(0·66–1·06)	··	0·78	(0·59–1·03)	··	··	··	··

	3	0·79	(0·68–0·92)	··	0·84	(0·70–0·99)	··	0·94	(0·72–1·23)	··	··	··	··

	High	0·75	(0·62–0·90)	··	0·73	(0·60–0·90)	··	1·20	(0·99–1·45)	··	··	··	··

Community beautification		··	··	··	··	··	··	··	··	··	0·0001	0·0001	<0·0001

	Trend	0·95	(0·91–0·98)	0·0021	0·92	(0·88–0·97)	0·0005	1·00	(0·95–1·05)	0·89	··	··	··

	Low	1·00	··	··	1·00	··	··	1·00	··	··	··	··	··

	2	1·07	(0·85–1·34)	··	0·87	(0·66–1·15)	··	1·22	(0·89–1·66)	··	··	··	··

	3	0·89	(0·71–1·12)	··	0·72	(0·51–1·02)	··	1·08	(0·83–1·40)	··	··	··	··

	4	0·99	(0·80–1·21)	··	0·84	(0·64–1·10)	··	1·06	(0·80–1·41)	··	··	··	··

	5	0·87	(0·72–1·05)	··	0·71	(0·55–0·91)	··	1·09	(0·85–1·40)	··	··	··	··

	High	0·75	(0·61–0·92)	··	0·63	(0·48–0·82)	··	0·99	(0·72–1·37)	··	··	··	··

Community disorder		··	··	··	··	··	··	··	··	··	0·022	0·13	<0·0001

	Trend	1·10	(1·03–1·17)	0·0042	1·09	(1·01–1·18)	0·035	1·16	(1·05–1·28)	0·0033	··	··	··

	Low	1·00	··	··	1·00	··	··	1·00	··	··	··	··	··

	2	1·01	(0·90–1·15)	··	1·09	(0·94–1·27)	··	1·00	(0·81–1·22)	··	··	··	··

	3	1·06	(0·92–1·22)	··	1·14	(0·95–1·36)	··	1·16	(0·92–1·46)	··	··	··	··

	High	1·48	(1·17–1·86)	··	1·39	(1·00–1·95)	··	1·68	(1·21–2·31)	··	··	··	··

Traffic density		··	··	··	··	··	··	··	··	··	<0·0001	0·0001	<0·0001

	Trend	0·98	(0·92–1·04)	0·42	0·85	(0·79–0·92)	0·0001	1·16	(1·06–1·27)	0·0009	··	··	··

	Low	1·00	··	··	1·00	··	··	1·00	··	··	··	··	··

	2	1·27	(1·05–1·52)	··	1·14	(0·79–1·64)	··	1·08	(0·88–1·32)	··	··	··	··

	3	1·27	(1·07–1·52)	··	0·92	(0·65–1·31)	··	1·48	(1·19–1·84)	··	··	··	··

	High	0·99	(0·81–1·20)	··	0·78	(0·55–1·11)	··	1·35	(0·98–1·86)	··	··	··	··

Bike lanes		··	··	··	··	··	··	··	··	··	0·060	0·0001	0·0036

	Absent	1·00	··	··	1·00	··	··	1·00	··	··	··	··	··

	Present	0·58	(0·45–0·73)	<0·0001	0·65	(0·48–0·87)	0·0040	0·38	(0·34–0·44)	<0·0001	··	··	··

Traffic signals		··	··	··	··	··	··	··	··	··	0·0003	0·049	0·20

	Absent	1·00	··	··	1·00	··	··	1·00	··	··	··	··	··

	Present	0·68	(0·52–0·89)	0·0055	0·76	(0·58–0·99)	0·046	0·47	(0·36–0·59)	<0·0001	··	··	··

Integrated built environment score		··	··	··	··	··	··	··	··	··	0·0007	<0·0001	<0·0001

	Trend	0·85	(0·78–0·91)	<0·0001	0·85	(0·77–0·93)	0·0007	0·87	(0·78–0·97)	0·015	··	··	··

	Lower quality	1·00	··	··	1·00	··	··	1·00	··	··	··	··	··

	2	0·80	(0·50–1·27)	··	0·84	(0·40–1·76)	··	0·77	(0·39–1·49)	··	··	··	··

	3	0·66	(0·41–1·07)	··	0·65	(0·31–1·36)	··	0·71	(0·36–1·39)	··	··	··	··

	Higher quality	0·58	(0·35–0·93)	··	0·60	(0·28–1·28)	··	0·58	(0·29–1·15)	··	··	··	··


The models include age, sex, education, household wealth, country income classification, urban–rural location, and a random intercept for communities. The trend is the RR for the linear association between a 1-unit increase in the built environment feature scales and obesity. RR=relative risk.

In adjusted analyses, higher-scoring built environments based on the integrated built environment score were associated with lower obesity overall (RR 0·58, 95% CI 0·35–0·93) than in lower-scoring environments (p=0·025). The categorical effects were less consistent in urban–rural stratified analyses, but statistically significant trends were observed overall (RR 0·85, 95% CI 0·78–0·91) and in urban (0·85, 0·77–0·93) and rural (0·87, 0·78–0·97) communities. Finally, traffic signal presence was associated with lower obesity rates across urban and rural communities (RR for overall association 0·68, 95% CI 0·52–0·89), with the association appearing more robust in rural areas (0·47, 0·36–0·59, p-heterogeneity <0·0001).

We found a strong indication that the associations between built environment features and obesity varied across country income levels and regions. Of the seven associations examined between built environment features and obesity, including the integrated built environment score, six showed significant heterogeneity across country income levels. The exception was the association between community disorder and obesity, consistent across country income levels (p-value for heterogeneity=0·13). Traffic density was not strongly associated with obesity overall. Still, subgroup analyses by country income levels reveal that traffic density was positively associated with obesity, although not statistically significantly, in LICs, but it was found to be inversely associated with obesity at higher country income levels. Analyses by region also showed significant heterogeneity in six of seven built environment-obesity associations. Here, traffic signals were consistently and inversely associated with obesity across regions. We show the stratified analyses of the association between integrated built environment scores and obesity across regions (appendix p 4). The integrated score was less consistently associated with obesity in individual regions, for example in south Asia, China, Africa, and North America, probably because of less statistical precision for subgroup analyses, but remained inversely associated in Russia and the former Soviet Republics and the Middle East. The integrated built environment score showed a trend for a positive association with obesity in South America (RR 1·07, 95% CI 1·01–1·14), and this was confirmed in the categorical analysis.

Of the analysed sample, 114 045 participants (79·6%) had physical activity and BMI data. Walking physical activity comes from the International Physical Activity Questionnaire, and the weekly mean walking MET-min was 1238 (SD 1847) overall, 1417 (2021) in males, and 1117 (1708) in females. We examined the distribution of physical activity forms by country income level and urban–rural location (appendix p 12). Household and job-related physical activity was more common in LICs and rural areas than in HICs and urban areas. Built environment features (eg, pedestrian safety features and the integrated built environment score) were associated with increased walking (appendix pp 5–6). Higher-quality built environment scores were associated with more activity by 427·55 MET-minutes per week (95% CI 250·30 to 604·81) compared with lower-scoring communities after adjusting for individual-level, household-level, community-level, and country-level covariates. Increasing levels of community disorder were associated with reduced MET-minutes from walking (b_trend_=–80·30 MET-minutes per week, 95% CI –143·33 to –17·27). In adjusted models, beautification, traffic density, bike lanes, and traffic signals were not independently associated with walking. In urban–rural stratified analyses, community beautification was associated with increased walking in urban communities (–70·95 MET-minutes per week, 22·20 to 119·70), as was the presence of traffic signals in rural areas (1106·86, 337·08 to 1876·63). When comparing urban and rural areas, three built environment features and the integrated score were associated with walking in urban areas, whereas only traffic signals were associated with walking in rural communities.

We analysed the relationship between built environment, physical activity, and obesity by including walking in regression models of built environment attributes and obesity. We examined the change in RR for each built environment attribute and obesity between models with and without walking (appendix p 7). Walking physical activity did not substantially attenuate the association between built environment attributes and obesity. For example, the association between the integrated built environment score and obesity was weakened by 0·5% (from a RR of 0·87 to 0·88) when walking was included in the model. Other attributes showed a similar magnitude of attenuation, varying between 0·1% and 0·9%. The magnitude of the RR of obesity for increasing traffic density was increased by 2·0% from 0·94 to 0·92 when walking physical activity was included as a covariate.

Correlations between built environment features identified in EPOCH photographs and walkability domains in the Neighbourhood Environment Walkability Scale showed moderate positive associations, varying between 0·34 for walking infrastructure and 0·52 for pedestrian safety with a correlation for the overall score of 0·39.

## Discussion

This study describes the association between built environment features captured in photographs and obesity in the PURE cohort of more than 140 000 individuals from 530 communities in 21 countries, with extensive coverage of low-income settings and rural areas. To the best of our knowledge, PURE is one of the most comprehensive studies to examine this issue. This study shows an application of our novel photograph method that correlates well with other direct built environment measures,^26^, ^28^ making it possible to examine built environment features across the diverse communities and countries involved in PURE. The analyses show that built environment features vary across communities, with some patterns emerging according to urban–rural locations that were consistent for LMICs but not HICs. Pedestrian safety features were less common in rural LMIC and LIC communities than in urban communities also in LMICs and LICs, but urban–rural differences were lacking in HICs. A similar pattern was found with features of community disorder. Natural features such as fields, forests, bodies of water, and mountains were more common in urban areas than rural areas, except in HICs, which had more natural features in rural areas. The integrated built environment score was significantly associated with obesity, as were several built environment attributes. Individually, pedestrian safety features, beautification, bike lanes, traffic signals, and community disorder were associated with lower obesity. In contrast, community disorder was positively associated with obesity after adjusting for important confounders.

Obesity continues to increase globally. The prevalence of obesity in this study was about three-fold higher in participants from HICs and UMICs than LMICs and LICs (30% *vs* 10%), a pattern consistent with previous reports that increased per capita gross domestic product is associated with greater obesity rates.^38^ Yet, in addition to the influence of country economics, these analyses show that built environment features, including walking and cycling infrastructure and beautifying features, could be associated with obesity rates. Differences in contextual factors between urban and rural communities and countries likely shape individual behaviour and interaction with the built environment. Although the causal pathway between the built environment and obesity is likely multifactorial, the environment could influence physical activity, diet, and other lifestyle factors related to obesity,^39^, ^40^ and our analyses suggest that the built environment-outcome associations might be more consistent in urban areas, particularly for walking as an intermediate outcome. However, more robust mediation analyses are required to confirm this association.

Few multicountry studies have examined the built environment's influence on health among older adults from LMICs. A systematic review of natural experiments of built environment changes and obesity-related outcomes found fewer studies examining obesity as an outcome (three studies) compared with nutrition or physical activity outcomes (18 and 17 studies, respectively);^41^ only one of the reported built environment change–obesity associations was statistically significant. The review found stronger effects on physical activity, particularly related to improvements in active transportation infrastructure. A separate systematic review of 21 studies, mainly from HICs and urban areas, found that access to bicycle lanes was associated with a 50% greater likelihood of physical activity among adolescents.^42^ However, existing research has been limited by the scarce inclusion of diverse international communities.

Measurement of the built environment has become increasingly important in public health as researchers have sought to understand its relationship with health outcomes. Many measures are available, including those based on questionnaires, audit tools, direct observation, or GIS-based measures.^10^, ^43^ Although each approach has strengths, no current tool offers a similar method to our photograph-based instrument. The method described here combines direct observation through photographs that can be scored for built environment features. As a result, rapid assessment of the built environment can be applied across diverse communities, including assessing rural and remote communities that have limited access to GIS-based built environment measures. In addition, the simple nature of this instrument allows for serial use in communities to assess built environment changes.

Our analyses found that an integrated built environment score showed strong associations with obesity, and walking attenuated only a small part of this association. However, this result should be interpreted carefully. Walking as exercise versus a method of mobility requires different amounts of energy and cardiometabolic function to reduce obesity. For example, high-pace walking requires almost triple the amount of METs compared with normal-pace walking.^44^ Walking under the sun or in hot conditions beyond the human thermal comfort zone (higher than 18°C to 26°C) might not be preferable for mobility for the general population who reside in tropical regions as it impairs cognitive-related tasks and increases the risk of heat-related illnesses.^45^, ^46^

There are differences in the levels and types of physical activity occurring in rural and urban environments, which affect how likely the built environment is to influence physical activity. Our data indicate that a smaller proportion of recreational physical activity occurs in rural areas, and such activity (eg, walking, running, and cycling) could be more sensitive to built environment features. Indeed, our analyses of participants with physical activity data suggested that associations between the built environment and walking were substantially more robust in urban areas than rural areas. This finding suggests that the built environment could have more influence on recreational physical activity compared with physical activity related to housework and occupations, which make up a larger share of physical activity reported in rural communities.^30^ Despite our adjustment strategy, confounding remains an issue and statistical adjustment cannot fully capture the potential socioeconomic confounding. An alternative interpretation is that residential selection bias might influence stronger associations in urban areas, making the associations between built environment features and obesity appear more pronounced in urban areas or high-income environments than rural areas or low-income settings.^47^, ^48^

Community disorder and, to a lesser extent, increased traffic density were associated with high rates of obesity. Community disorder was positively associated with obesity in urban and rural areas, although the magnitude of association was stronger in urban communities. Community disorder was, however, consistently associated with obesity across country income levels and geographical regions. The associations with increasing traffic density showed heterogeneity and were positive in rural areas and negative in urban areas. A positive relationship between traffic and population density could explain the association in urban areas. Although we did not directly measure population density, the mechanism could be attributed to increased walking and decreased reliance on motorised transport.^49^ In rural areas, increasing traffic density could be related to higher relative prosperity of an area, indicated by the presence of more cars. It could also indicate changes in transportation patterns away from walking and toward cars or other modes of transport, reducing the amount of physical activity.

The analyses have limitations; while the EPOCH photographs and the EP-NET instrument have strengths in their simplicity, the photo evaluation is a small snapshot of each community's environment and is done at a single target location. The target location or start point is typically a central commercial area within a community. It might overestimate walkability, particularly in communities that are not densely built throughout, and the photos can be affected by the season, weather, and time of day. To address this, we collected the photographs in good weather and during the daytime to achieve the best representation of the built environment. Overall, EPOCH photographs and the EP-NET scoring correlated reasonably well with other direct measures of the built environment and neighbourhood walkability scales (eg, the Neighbourhood Environment Walkability Scale) we collected,^26^, ^37^ but can be more easily collected without involving individual respondents.^28^

Other limitations of the study that are important to note include the cross-sectional design. We do not have a temporal separation between our photo data collection and outcome assessment in this initial analysis. Although many comparisons are made between built environment features and outcomes, these are framed as exploratory rather than causal, given the design limitations. In time, however, we plan to repeat built environment assessments on some PURE communities, allowing longitudinal comparisons between built environment and BMI changes. We will examine how the built environment and the food environment of communities affect dietary outcomes. PURE recruited participants from selected communities of the world, intentionally chosen for diversity, but not a strictly representative sample of communities.^32^ This design could affect the prevalence estimates, but we do not anticipate it will affect the built environment-outcome associations. Finally, we define obesity using BMI, which has limitations, such as low sensitivity in identifying adiposity.^50^ In this study, we find that using BMI is acceptable as it is available in all PURE participants, and the paper was internationally comparative. In addition, study results were not substantially changed by using different cutoffs to define obesity.

The study shows that an integrated built environment score and distinct features of the built environment, assessed using community photographs, including bike lanes, pedestrian safety, community disorder, and traffic density, are associated with obesity. In this unique and diverse sample, urban–rural and between-country heterogeneity was shown in these associations, suggesting that the effect of the built environment on obesity could be context-specific. Further investigation of the built environment in diverse communities involving LMICs and examination of longitudinal relationships, that is, the effect of the built environment over time, are needed to enable a greater understanding and ability to influence our environment to affect health positively.

### PURE Study staff members

### Contributors

### Data sharing

The Population Health Research Institute (PHRI) is the sponsor of this study. The PHRI believes that the dissemination of research results is vital and the sharing of data is important. PHRI prioritises access to data to researchers who have worked on the PURE study for a substantial duration, have played major roles, and have participated in raising the funds to conduct the study. Data will be disclosed to these individuals upon request for specific proposals after review and approval of the proposed use of the data by a PURE Review Committee. Specific collaborative projects can be developed with external groups with similar data for joint analyses as long as there is an exchange of data and the proposal enhances the findings from PURE. The underlying data for this clinical study contains personal information and personal health information of participants who were involved, which is protected under Canada's privacy laws, the Health Insurance Portability and Accountability Act of 1996 (USA), and General Data Protection Regulation, among other international laws governing privacy. Consent for public disclosure of this information was not obtained and could pose a threat to confidentiality and violate privacy laws. Therefore, sharing of individual data is usually not possible, except for with PURE investigators. PHRI has no objection to sharing the information under confidentiality and with appropriate data protection and privacy. PHRI follows this procedure and does not share or link data from clinical studies publicly when such data are or contain personal health information. Requests for access to information can be sent to the PURE Publications Committee and the PHRI study project office (phri.contracts@phri.ca).

**This online publication has been corrected. The corrected version first appeared at thelancet.com/lancetgh on October 16, 2024**

## Declaration of interests

CKC is supported by a National Health and Medical Research Council of Australia Investigator grant (APP1195326). AR declares institutional support from the Swedish Research Council, the Swedish Heart and Lung Foundation, and grants from the Swedish state under the agreement concerning research and the education of doctors. LMP-V declares a research grant from the Philippine Council for Health Research and Development, Department of Science and Technology. PH declares institutional grants from the US National Institute of Environmental Health Sciences (R21ES031226) and the National Heart, Lung, and Blood Institute (R01HL50119). All other authors declare no competing interests.

## Footnotes

† Features are scaled within the integrated Built Environment Score; disorder and traffic density are reverse-coded.

## Footnotes

* Higher maximum scores are possible with increasing quality of environment.
