# Correction to *Lancet Glob Health* 2024; 12: e1794–806

**DOI:** 10.1016/S2214-109X(24)00426-1

**Published:** 2024-10-16

*Corsi DJ, Marschner S, Lear S, et al. Assessing the built environment through photographs and its association with obesity in 21 countries: the PURE Study.* Lancet Glob Health *2024; **12:** e1794–806*—This Article should have been published under a CC BY Open Access license. This correction has been made as of Oct 16, 2024.

