# Supplementary material for: Assessing the built environment through photographs and its association with obesity in 21 countries: the PURE Study
Source: Lancet Glob Health. 2024 Sep 27;12(11):e1794–806. doi: 10.1016/S2214-109X(24)00287-0 (PMC11483223; doi:10.1016/S2214-109X(24)00287-0)

THE LANCET
Global Health

Supplementary appendix

This appendix formed part of the original submission and has been peer reviewed. We post it as supplied by the authors.

Supplement to: Corsi DJ, Marschner S, Lear S, et al. Assessing the built environment through photographs and its association with obesity in 21 countries: the PURE Study. *Lancet Glob Health* 2024; published online Sept 27. https://doi.org/10.1016/S2214-109X(24)00287-0.

SUPPLEMENTAL APPENDIX

Corsi et al., "Assessing the Built Environment Through Photographs and Its Association With Obesity in 21 Countries: The PURE Study."

TABLE OF CONTENT

Supplemental Table 1 Number of individuals and communities by country and urban rural location, the PURE Study 2

Supplemental Table 2 Prevalence of Obesity (BMI greater than 30 kg/m^2) by Participant Characteristics across Country Income level and Urban-Rural location 3

Supplemental Table 3 Associations Between Integrated Built Environment Scores and Obesity (BMI greater than 30 kg/m^2); Fully-Adjusted Multilevel Poisson Regression Models, Region-specific models 4

Supplemental Table 4 Associations Between Community Built Environment Features and Walking (MET-minutes/week) From Fully-Adjusted Multilevel Regression Models, N= 118,843 5

Supplemental Table 5 Attenuation in the Association between Physical Environment Features and Obesity after including physical activity from walking as a covariate expressed as a percentage, N=112,341 7

Supplemental Figure 1 Directed acyclic graph depicting hypothesized relationships between built environment attributes and obesity, showing the role of confounders and covariates, PURE study 8

Supplemental Figure 2 Density plots of the body mass index distribution in urban (blue) and rural (red) samples of the PURE study by country 9

Supplemental Figure 3 Presence of natural features across urban and rural communities, by country-level income, N=530 communities in PURE 10

Supplemental Figure 4 Pearson Correlations between built environment features and the integrated BE score, overall and by urban and rural location 11

Supplemental Figure 5 Percentage of total MET-minutes by activity category, by country income level and urban-rural location, PURE, N=119,417 12

Supplemental Table 1 Number of individuals and communities by country and urban rural location, the PURE Study

	Communities (n)			Individuals (n)		
**Country**	**Total**	**Urban**	**Rural**	**Total**	**Urban**	**Rural**
**PURE**	**530**	**290**	**240**	**143,338**	**78,195**	**65,143**
India	78	32	46	25,662	12,915	12,747
China	94	39	55	39,779	20,051	19,728
Malaysia	34	20	14	12,480	4,770	7,710
Philippines	5	2	3	4,865	2,472	2,393
South Africa	9	5	4	2,818	1,440	1,378
Tanzania	6	3	3	1,674	979	695
Zimbabwe	3	1	2	819	251	568
Canada	69	44	25	10,228	7,146	3,082
Sweden	23	20	3	3,903	3,004	899
Poland	3	1	2	1,367	1,197	170
Turkey	38	25	13	4,031	2,618	1,413
Iran	20	11	9	5,971	3,007	2,964
UAE	3	1	2	1,290	818	472
Palestine	21	12	9	1,041	597	444
Saudi Arabia	17	16	1	1,442	1,387	55
Argentina	20	6	14	7,461	3,592	3,869
Brazil	13	7	6	5,507	3,618	1,889
Colombia	48	34	14	5,897	3,591	2,306
Chile	5	2	3	3,219	2,519	700
Russia	13	5	8	1,596	1,089	507
Kazakhstan	8	4	4	2,288	1,134	1,154

Supplemental Table 2 Prevalence of Obesity (BMI greater than 30 kg/m^2) by Participant Characteristics across Country Income level and Urban-Rural location

	High Income				Upper Middle Income				Lower Middle Income				Low Income			
	Urban		Rural		Urban		Rural		Urban		Rural		Urban		Rural	
**Characteristic**	**% Obese**	**95%CI**	**% Obese**	**95%CI**	**% Obese**	**95%CI**	**% Obese**	**95%CI**	**% Obese**	**95%CI**	**% Obese**	**95%CI**	**% Obese**	**95%CI**	**% Obese**	**95%CI**
**Overall**	26.3	(25.6,27.1)	30.8	(29.5,32.2)	34.8	(34.2,35.5)	29.5	(28.9,30.2)	12.6	(12.3,13.0)	9.3	(9.0,9.7)	14.3	(13.7,14.9)	4.7	(4.3,5.0)
Sex																
Female	26.6	(25.5,27.7)	30.8	(29.0,32.7)	39.2	(38.4,40.1)	33.8	(32.9,34.7)	14.0	(13.5,14.5)	11.1	(10.6,11.5)	19.4	(18.5,20.3)	6.5	(6.0,7.0)
Male	26.0	(24.9,27.2)	30.8	(28.8,32.9)	27.4	(26.4,28.4)	23.3	(22.3,24.2)	10.6	(10.1,11.2)	6.9	(6.4,7.4)	7.6	(6.9,8.3)	2.1	(1.7,2.5)
Age																
Less than 45 y	26.2	(24.7,27.8)	30.4	(27.5,33.4)	30.6	(29.4,31.8)	26.8	(25.6,28.0)	11.4	(10.7,12.1)	9.3	(8.7,9.9)	13.4	(12.5,14.3)	4.3	(3.8,4.9)
45-64 y	26.4	(25.4,27.3)	31.2	(29.5,32.9)	36.2	(35.4,37.1)	31.4	(30.6,32.3)	13.6	(13.1,14.1)	9.7	(9.2,10.1)	15.4	(14.6,16.3)	5.3	(4.8,5.8)
65 y and above	26.5	(24.2,28.9)	30.0	(26.4,33.8)	37.4	(35.5,39.4)	26.9	(25.1,28.9)	10.6	(9.6,11.6)	6.9	(5.9,8.1)	12.2	(10.6,13.9)	3.3	(2.5,4.3)
Education																
Trade/Coll/Univ	21.5	(20.6,22.4)	28.0	(26.1,30.0)	28.1	(27.0,29.2)	29.2	(27.2,31.2)	9.7	(9.1,10.3)	10.3	(8.7,12.1)	15.1	(13.9,16.4)	7.5	(5.3,10.2)
Sec/HigherSec	30.2	(28.7,31.8)	30.6	(28.4,33.0)	32.9	(31.8,34.0)	27.0	(25.8,28.3)	11.4	(10.9,12.0)	7.2	(6.8,7.7)	13.4	(12.5,14.2)	6.6	(5.8,7.4)
None/Prim/Unk	45.9	(43.2,48.7)	38.6	(35.2,42.0)	42.0	(40.9,43.1)	30.6	(29.8,31.4)	18.9	(18.0,19.9)	10.9	(10.4,11.4)	15.1	(14.0,16.1)	3.7	(3.4,4.1)

Supplemental Table 3 Associations Between Integrated Built Environment Scores and Obesity (BMI greater than 30 kg/m^2); Fully-Adjusted Multilevel Poisson Regression Models**, Region-specific models**

	South Asia			China			SE Asia			Russia & FSR		
	**RR**	**95%CI**	**p-value**	**RR**	**95%CI**	**p-value**	**RR**	**95%CI**	**p-value**	**RR**	**95%CI**	**p-value**
Integrated BE Score												
Trend	1.00	(0.92 , 1.09)	0.977	0.99	(0.90 , 1.10)	0.889	1.03	(0.95 , 1.13)	0.458	0.95	(0.92 , 0.99)	0.012
Lower quality	1.00			1.00			1.00			1.00		
2	1.38	(1.04 , 1.82)	0.024	1.33	(1.02 , 1.73)	0.033	1.00	(0.79 , 1.26)	0.985	0.67	(0.50 , 0.90)	0.008
3	0.94	(0.64 , 1.37)	0.737	1.08	(0.78 , 1.51)	0.642	1.08	(0.89 , 1.31)	0.453	0.85	(0.74 , 0.98)	0.022
Higher Quality	1.03	(0.79 , 1.36)	0.820	0.96	(0.70 , 1.32)	0.798	1.08	(0.80 , 1.47)	0.614	0.87	(0.77 , 0.98)	0.019
	**Africa**			**N. America/EU**			**Middle East**			**South America**		
	**RR**	**95%CI**	**p-value**	**RR**	**95%CI**	**p-value**	**RR**	**95%CI**	**p-value**	**RR**	**95%CI**	**p-value**
Integrated BE Score												
Trend	0.99	(0.90 , 1.09)	0.898	1.03	(0.94 , 1.13)	0.517	0.89	(0.82 , 0.97)	0.005	1.07	(1.01 , 1.14)	0.019
Lower quality	1.00			1.00			1.00			1.00		
2				0.85	(0.54 , 1.34)	0.482	0.99	(0.80 , 1.21)	0.916	0.94	(0.79 , 1.11)	0.438
3	0.98	(0.77 , 1.23)	0.834	0.97	(0.62 , 1.54)	0.909	0.74	(0.60 , 0.92)	0.007	1.14	(0.97 , 1.35)	0.119
Higher Quality	1.01	(0.70 , 1.46)	0.956	0.96	(0.61 , 1.51)	0.855	0.76	(0.56 , 1.02)	0.067	1.17	(0.97 , 1.41)	0.097

Supplemental Table 4 Associations Between Community Built Environment Features and Walking (MET-minutes/week) From Fully-Adjusted Multilevel Regression Models, N= 118,843

	Overall				Urban				Rural				Interaction p-values		
**Built Environment Features**	**Estimate**	**95%CI**		**p-value**	**Estimate**	**95%CI**		**p-value**	**Estimate**	**95%CI**		**p-value**	**Urban-rural**	**Country income**	**Region**
Pedestrian Safety													0.875	0.001	0.061
Trend	76.59	(14.33 ,	138.84)	0.016	84.36	(14.25 ,	154.48)	0.018	-36.24	-(178.45 ,	105.97)	0.617			
Low	0.00				0.00				0.00						
2	229.24	(4.27 ,	454.21)	0.046	240.34	-(26.62 ,	507.31)	0.078	92.02	-(332.54 ,	516.57)	0.671			
3	265.49	(89.28 ,	441.69)	0.003	288.37	(91.15 ,	485.59)	0.004	-89.80	-(539.02 ,	359.42)	0.695			
High	126.25	-(84.37 ,	336.87)	0.240	147.37	-(90.09 ,	384.83)	0.224	-122.64	-(576.94 ,	331.65)	0.597			
Community beautification													0.017	0.004	0.341
Trend	12.07	-(23.32 ,	47.45)	0.504	70.95	(22.20 ,	119.70)	0.004	-56.12	-(104.39 ,	-7.85)	0.023			
Low	0.00				0.00				0.00						
2	-6.03	-(228.43 ,	216.36)	0.958	106.58	-(246.76 ,	459.91)	0.554	-72.73	-(341.69 ,	196.23)	0.596			
3	44.17	-(173.11 ,	261.45)	0.690	223.52	-(109.63 ,	556.66)	0.189	-108.22	-(381.62 ,	165.19)	0.438			
4	-26.68	-(241.65 ,	188.29)	0.808	198.10	-(104.55 ,	500.74)	0.200	-247.20	-(554.61 ,	60.21)	0.115			
5	62.01	-(125.56 ,	249.57)	0.517	295.63	(19.57 ,	571.70)	0.036	-123.61	-(372.61 ,	125.38)	0.331			
High	45.33	-(158.10 ,	248.76)	0.662	385.54	(95.73 ,	675.35)	0.009	-382.15	-(668.33 ,	-95.96)	0.009			
Community Disorder													0.429	0.604	0.543
Trend	-80.30	-(143.33 ,	-17.27)	0.013	-127.63	-(213.38 ,	-41.88)	0.004	28.17	-(68.22 ,	124.56)	0.567			
Low	0.00				0.00				0.00						
2	-89.20	-(236.17 ,	57.76)	0.234	-90.70	-(276.53 ,	95.12)	0.339	-73.34	-(304.91 ,	158.22)	0.535			
3	-189.77	-(349.47 ,	-30.06)	0.020	-235.89	-(446.65 ,	-25.13)	0.028	2.07	-(247.09 ,	251.22)	0.987			
High	-212.18	-(426.19 ,	1.84)	0.052	-394.84	-(709.38 ,	-80.30)	0.014	94.82	-(206.82 ,	396.45)	0.538			
Traffic Density													0.316	0.143	0.309

Trend	30.80	-(33.40 ,	95.00)	0.347	33.04	-(63.76 ,	129.85)	0.503	1.33	-(87.00 ,	89.67)	0.976			
Low	0.00				0.00				0.00						
2	-39.14	-(226.44 ,	148.16)	0.682	-196.48	-(722.90 ,	329.94)	0.464	-24.91	-(226.83 ,	177.01)	0.809			
3	52.71	-(133.31 ,	238.74)	0.579	-142.41	-(652.32 ,	367.49)	0.584	112.32	-(102.98 ,	327.63)	0.307			
High	58.26	-(148.13 ,	264.65)	0.580	-90.76	-(602.63 ,	421.10)	0.728	-184.64	-(517.67 ,	148.40)	0.277			
Bike Lanes													0.641	0.538	0.107
Absent	0.00				0.00				0.00						
Present	106.41	-(202.03 ,	414.85)	0.499	104.28	-(213.74 ,	422.30)	0.520	-328.40	-(1624.33 ,	967.53)	0.619			
Traffic Signals													0.058	0.166	0.254
Absent	0.00				0.00				0.00						
Present	280.20	-(136.00 ,	696.40)	0.187	66.58	-(419.88 ,	553.05)	0.788	1106.86	(337.08 ,	1876.63)	0.005			
Integrated BE Score													<0.001	<0.001	0.047
Trend	142.31	(85.90 ,	198.71)	<0.001	212.68	(141.19 ,	284.17)	<0.001	34.86	-(51.84 ,	121.55)	0.431			
Lower quality	0.00				0.00				0.00						
2	221.49	(68.75 ,	374.23)	0.004	223.66	(4.72 ,	442.59)	0.045	236.74	(31.11 ,	442.38)	0.024			
3	379.89	(220.77 ,	539.00)	<0.001	478.38	(262.00 ,	694.76)	<0.001	260.81	(34.49 ,	487.13)	0.024			
Higher Quality	427.55	(250.30 ,	604.81)	<0.001	632.11	(398.85 ,	865.38)	<0.001	44.34	-(225.71 ,	314.39)	0.748			

Models are adjusted for age, sex, education, household wealth, country income classification, urban-rural location, and a random intercept for communities.

Supplemental Table 5 Attenuation in the Association between Physical Environment Features and Obesity after including physical activity from walking as a covariate expressed as a percentage, N=112,341

	Adjusted Model				Adjusted Model + Walking				
**Physical Environment Features**	**RR_1**	**95%CI**		**p-value**	**RR_2**	**95%CI**		**p-value**	**Attenuation (%)**
Pedestrian Safety	0.91	(0.86 ,	0.96)	0.001	0.91	(0.86 ,	0.96)	0.001	-0.3
Community beautification	0.95	(0.92 ,	0.99)	0.007	0.95	(0.92 ,	0.99)	0.007	-0.1
Community Disorder	1.09	(1.02 ,	1.16)	0.012	1.08	(1.02 ,	1.15)	0.015	-0.3
Bike Lanes	0.60	(0.48 ,	0.76)	<0.001	0.60	(0.48 ,	0.76)	<0.001	-0.5
Traffic Signals	0.67	(0.52 ,	0.87)	0.003	0.68	(0.53 ,	0.88)	0.003	-0.9
Integrated BE Score	0.87	(0.82 ,	0.93)	<0.001	0.88	(0.82 ,	0.93)	<0.001	-0.5
Traffic Density	0.94	(0.88 ,	1.00)	0.044	0.92	(0.85 ,	1.00)	0.042	2.0

BE exposures were modelled continuously as a linear trend. Attenuation in the association between physical environment features and obesity is calculated after adjusting multilevel regression models for physical activity from walking. The formula for attenuation, $\%=(1-RR_2/RR_1)\times100$, is used, with attenuation in the RR expressed as a percentage. RR_2 is the RR from the adjusted model, including walking, and RR_1 is from the adjusted model without walking. Both models include age, sex, education, household wealth, country income classification, urban-rural location, community SES, and a random intercept for communities.

Supplemental Figure 1 Directed acyclic graph depicting hypothesized relationships between built environment attributes and obesity, showing the role of confounders and covariates, PURE study

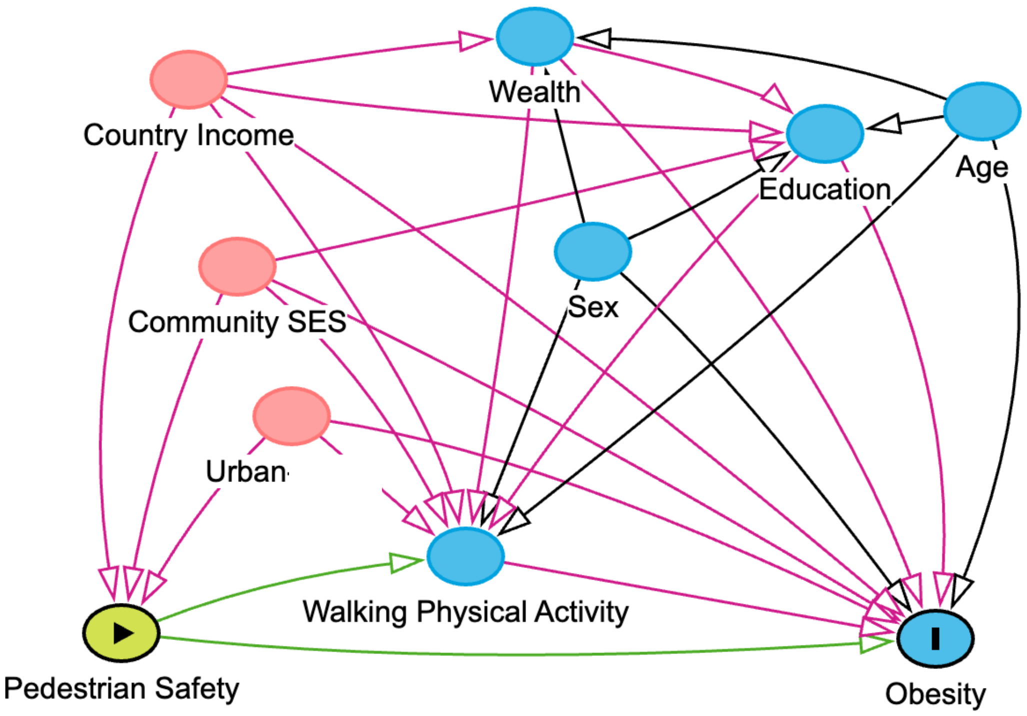

SES, socioeconomic status

Pedestrian safety is shown as an example of a built environment attribute. Other built environment attributes modelled with this framework include community beautification, community disorder, traffic density, bike lanes, traffic signals, and the integrated built environment score.

Supplemental Figure 2 Density plots of the body mass index distribution in urban (blue) and rural (red) samples of the PURE study by country

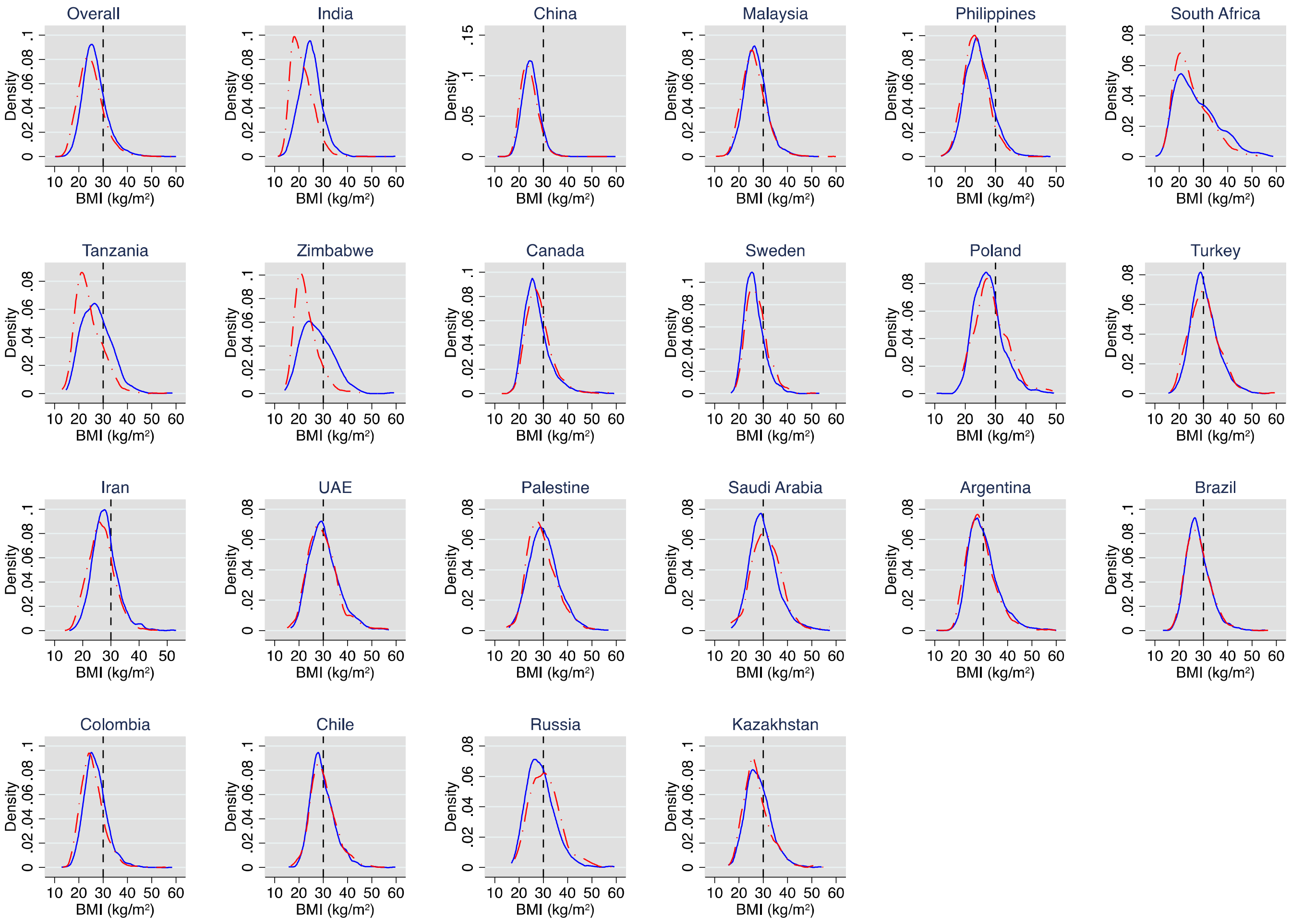

Supplemental Figure 3 Presence of natural features across urban and rural communities, by country-level income, N=530 communities in PURE

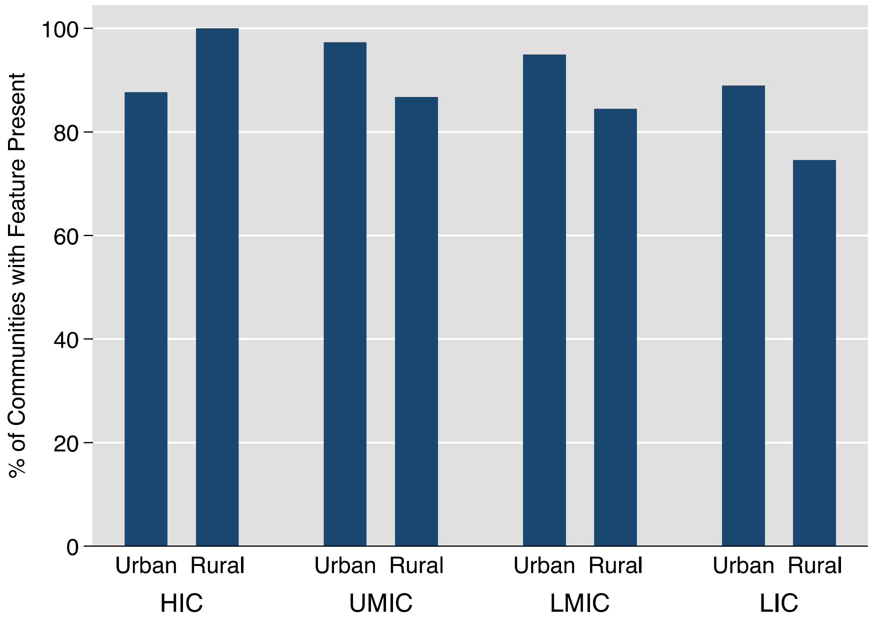

Natural Features include bodies of water, mountains, greenbelts, fields, forests or deserts.

Supplemental Figure 4 Pearson Correlations between built environment features and the integrated BE score, overall and by urban and rural location

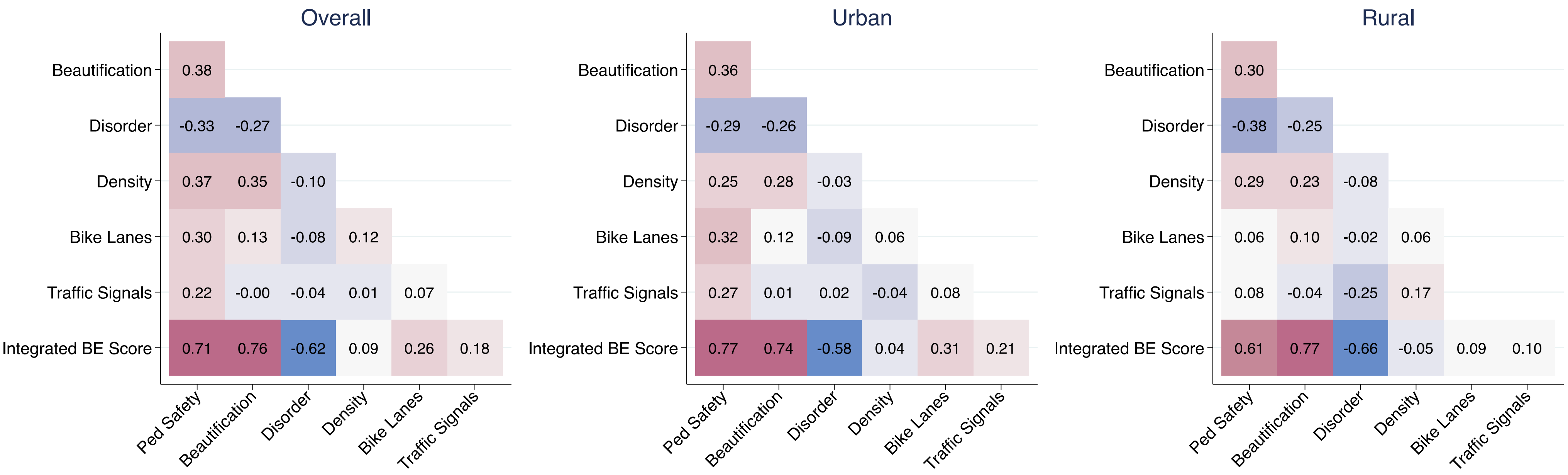

Supplemental Figure 5 Percentage of total MET-minutes by activity category, by country income level and urban-rural location, PURE, N=119,417

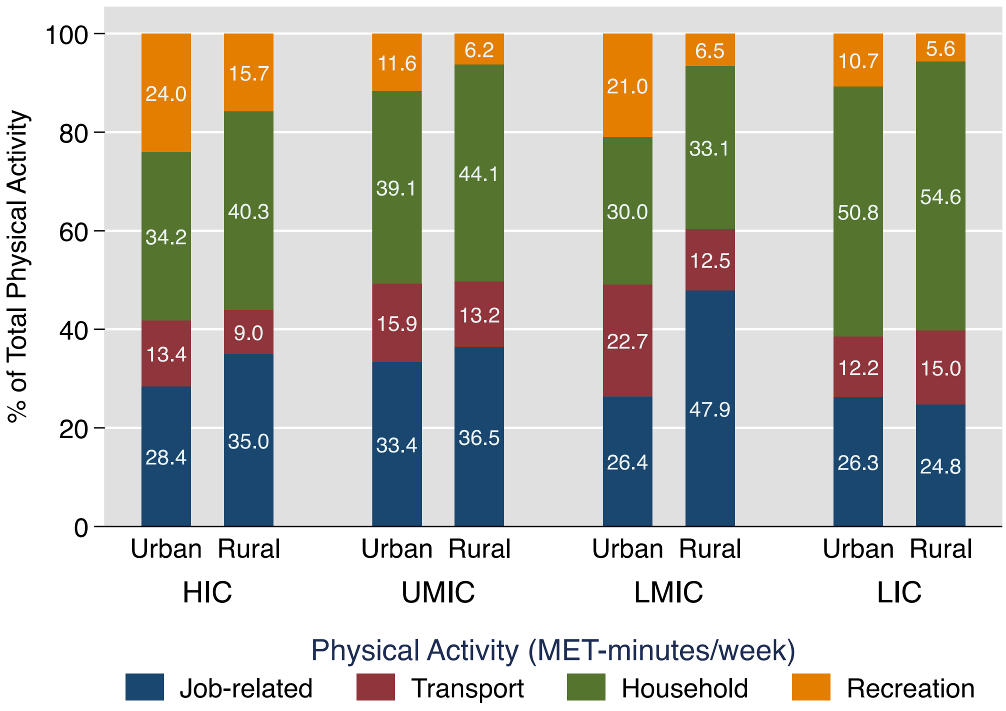

Supplement: Supplementary appendix [file mmc1.pdf]
